# Prevalence of chronic kidney disease in patients with type 2 diabetes in Spain: PERCEDIME2 study

**DOI:** 10.1186/1471-2369-14-46

**Published:** 2013-02-22

**Authors:** Antonio Rodriguez-Poncelas, Josep Garre-Olmo, Josep Franch-Nadal, Javier Diez-Espino, Xavier Mundet-Tuduri, Joan Barrot-De la Puente, Gabriel Coll-de Tuero

**Affiliations:** 1 EAP Anglès, Girona, Spain; 2 Unitat de Recerca IAS, Salt, Spain; 3 EAP Raval Sud, Barcelona, Spain; 4 Unitat de Recerca Jordi Gol, Barcelona, Spain; 5 EAP Tafalla, Navarra, Spain; 6 EAP El Carmel, Barcelona, Spain; 7 EAP Salt, Girona, Spain

**Keywords:** Cross-sectional study, Chronic kidney disease, Renal impairment, Albuminuria, Type 2 diabetes mellitus

## Abstract

**Bakground:**

The objective of this study was to determinate the prevalence of chronic kidney disease (CKD) and the different stages of CKD in patients with type 2 diabetes mellitus (DM2) treated in primary care consults in Spain.

**Methods:**

A national cross-sectional study was performed in primary care consults. The following data were collected: demographic and anthropometric information; list of present cardiovascular risk factors (CVRF); previous macrovascular and microvascular disease history; physical examination and analytical data from the previous 12 months, including the urine albumin-creatinine ratio (UACR) and estimated glomerular filtration rate (eGFR) to evaluate renal function.

**Results:**

With regard to the patients, 27.9% presented some degree of CKD as follows: 3.5% with stage 1; 6.4% with stage 2; 16.8% with stage 3 (11.6% with stage 3A and 5.2% with stage 3B); and 1.2% with stages 4 and 5. The prevalence of patients with UACR ≥ 30 mg/g was 15.4% (13% microalbuminuria and 2.4% macroalbuminuria). Renal impairment (RI) was found in 206 patients (18%) of whom 133 patients (64.6%) was stage 3A, 60 patients (29.1%) was stage 3B and 13 patients (6.3%) stages 4 and 5. Among patients with RI, 143 patients (69.4%) had normoalbuminuria.

The following variables were significantly associated with CKD: age; sex (women); systolic arterial blood pressure (SABP) ≥ 150 mmHg; and a previous history of cardiovascular disease.

**Conclusions:**

The results showed that the prevalence for any type of CKD was 27.9%. A systematic determination of UACR and eGFR may contribute to an early diagnosis, thus allowing intervention during the initial stages of the disease when treatment is more efficient.

## Background

Chronic kidney disease (CKD) is considered an important public health problem [1], as it increases the mortality risk for any cause, which increases the frequency of cardiovascular disease episodes and the progression to end-stage renal disease (ESRD) independently of traditional CVRF [2-5].

Diabetes mellitus (DM) is one of the leading causes of CKD and is recognised as one of the leading causes of ESRD in the United States [6]. Approximately 40% of the adult population diagnosed or not diagnosed with DM has some degree of CKD in the United States. CKD increases the cost of managing DM [7]. CKD commonly initiates at the prediabetes stage due to its coexistence with other CVRF, and CKD is present in one-third of DM2 patients [8].

Annual screening for CKD in DM2 patients, including the UACR and eGFR, is recommended at the moment of disease diagnosis. Early identification of CKD would allow immediate intervention, thus diminishing the progression of renal disease and cardiovascular risk. Chronic Kidney Disease include diminished eGFR and/or increased UACR, although a considerable percentage of DM2 patients present reduced eGFR without increased UACR [9].

The guidelines provided by the National Kidney Foundation (NKF) for the evaluation, classification, and stratification of CKD in the Kidney Disease Outcomes Quality Initiative (KDOQI) [10] define the following as diagnostic criteria for CKD: a eGFR value below 60 ml/min/1.73 m^2^ in a time period equal or superior to three months or the presence of renal lesion with or without reduced GFR in a time period equal or superior to three months. The concept of renal lesion is defined as the presence of structural or functional alterations of the kidney detected directly by histological alteration in the renal biopsy or indirectly by an UAE increase, alterations in the urine sediment, or differences identified with imaging techniques. The combination of these clinical criteria is the basis for the classification of CKD in five stages.

The objective of this study was to establish the prevalence of CKD and the different stages of CKD in patients with DM2 treated in primary care consults in Spain.

## Methods

### Study design and population

This study was a national cross-sectional study performed in primary care consults. Estimating a higher prevalence of chronic kidney disease in women than in men, and higher in those over 64 years, there was a proportional stratified sampling. A sample size of 1153 patients was calculated assuming a 0.05 alpha error and 2.5% precision for an estimated prevalence of 25%. Foreseeing a 20% loss due to erroneous or incomplete data, the size of the sample was increased to 1383 patients. During the recruitment, 1279 patients were included in the study, but 134 (10.4%) patients were discarded because of incomplete or erroneous data. To rule out a bias attributable to patients discarded we compared each other groups, of all variables compared did not show differences. The total number of valid patients for the final analysis was 1145. The patients included both genders and were over 40 years old. Moreover, the patients were diagnosed with DM2, and they had all of the variables necessary for the study included in their clinical history. Each researcher included 15 patients who met the inclusion criteria, a maximum of 3 patients per day. The patients were selected by convenience sample of the first 3 DM2 patients each day who came to the consult for any reason and met the inclusion criteria until complete the number of participants per researcher.

The information for each participant was collected in the period from February to July 2011. The Ethical and Clinical Investigation Committee of the Institut d’ Assistència Sanitària (IAS) in Salt, Girona, Spain approved the study. To obtain the necessary data for this study from the clinical history of patients, all participants provided written informed consent.

### Variables and procedure

The following variables were collected during the visit: age, gender, ethnicity, weight, height, body mass index (BMI) was calculated as weight in kilograms divided in meters squared; obesity was defined as BMI ≥ 30 kg/m^2^, abdominal waist circumference, CVRF. Cardiovascular diseases (CVD) was collected through the medical record as a history of Stroke (ischaemic cerebrovascular disease) included only symptomatic brain infarction, and did not include silent brain infarction, transient ischaemic attack or brain haemorrhage. CHD included a previous history of myocardial infarction, angina pectoris, the presence of coronary interventions or the presence of ECG abnormalities suggestive of CHD. PAD was diagnosed by an ankle-brachial pressure index of < 0.9 and/or two absent foot pulses. Diabetes mellitus was defined as fasting glucose ≥ 126 mg/dL or non-fasting glucose ≥ 200 mg/dL or use of glucose-lowering drugs. Hypertension was defined as SBP of 140 mmHg or greater, DBP of 90 mmHg or greater, or use of antihypertensive medications irrespective of BP. Hyperlipidemia was defined as total cholesterol ≥ 250 mg/dL, LDL cholesterol > 155 mg/dL, HDL cholesterol < 40 mg/dL in men and < 48 mg/dL in women, trigycreides > 150 mg/dL or pharmacologig lipid lowering treatment. Cigarette smoking was defined as never/past/current.

The following clinical and analytical measurements were collected: DM2 duration, arterial pressure (average of the last three determinations), drugs taken by the patient at the time the data were collected (antidiabetic, antihypertensives, hypolipemiant, antiplatelet, and anticoagulants). Basal glycaemia, glycosylated haemoglobin (HbA1c), plasmatic haemoglobin, total cholesterol, LDL cholesterol, HDL cholesterol, non-HDL cholesterol, triglycerides, serum creatinine after overnight fasting for at least 12 hr, and the urine albumin-creatinine ratio (UACR) in a urine sample collected in the first morning urine specime, using the most recent value of the last 12 months. The analysis determination was not performed by a single laboratory.

In this study, the presence of CKD was based on KDOQI [10] criteria as follows: patients with GFR < 60 ml/min/1.73 m^2^ or the presence of renal damage if UACR values were elevated (UACR ≥ 30 mg/g). Considering that all of the values for serum creatinine were measured using the modified kinetic method of Jaffe, the MDRD-IDMS (Modification of Diet in Renal Disease study- isotope dilution mass spectrometry) equation [11] was used to calculate the GFR. Albuminuria was defined as a UACR of 30 mg/g or more. Microalbuminuria was defined as a UACR ranging from 30 to 299 mg/g, and macroalbuminuria was defined as a UACR of 300 mg/g or more. CKD was defined as the presence of albuminuria or eGFR < 60 mL/min/1.73 m^2^. Stages of CKD were defined as follows: stage 1 (eGFR ≥ 90 mL/min/1.73 m^2^ and UACR ≥30 mg/g); stage 2 (eGFR 60–89 mL/min/1.73 m^2^ and UACR ≥30 mg/g); stage 3 (eGFR 30–59 mL/min/1.73 m^2^ regardless of UACR); stage 4 (eGFR 15–29 mL/min/1.73 m^2^ regardless of UACR); and stage 5 (eGFR <15 mL/min/1.73 m^2^ regardless of UACR). Two of three determinations of eGFR or/and UACR, in a period of three or more months, should be presented normal or altered values. The cases that did not fulfil this requisite were excluded from the final analysis of the study. The same requirement of two or more measurement in three months was applied to a classification of presence CKD or absence CKD (“no CKD”).

#### Statistical analysis

Measurements of central tendency and dispersion were used to perform a descriptive analysis of the quantitative variables studied. Absolute and relative frequencies were used for qualitative variables. The global CKD prevalence was calculated, and the CKD prevalence of each stage was also calculated. Age group and sex stratified the results. Bivariate analysis of contrasting hypotheses was used to establish the presence of statistically significant differences in the clinical characteristics of the patients depending on the presence or absence of CKD and its stage. Proportion comparison was performed by means of a Chi-squared test with Yates correction when it was considered necessary. The means of the clinical and analytical variables were compared with the ANOVA test and the medians were compared with the the Kruskal-Wallis test depending on the distribution of the data. The normal distribution of continuous variables was contrasted with the Shapiro-Wilk test. The Bonferroni correction was used for multiple testing. The association degree between the demographic and clinical characteristics of the patients and the presence or absence of CKD was established with a binomial logistic regression. The results were expressed as absolute numbers, percentages, medians, standard deviations, odd ratios, and CI of 95%. Statistical significance was set at 0.05 when contrasting hypotheses. Data analysis and processing were performed using the SPSS 14.0 statistical program for Windows.

## Results

Table 1 shows the clinical and demographic characteristics of the 1145 participants. When comparing men and women, no significant differences were detected with age, HbA1c levels, and arterial pressure. Men presented higher values for creatinine, because men have more muscle mass than women, and smoking than women. Total cholesterol levels and HDL cholesterol were higher in women than in men. The prevalence of coronary disease, ictus, and peripheral vascular disease was higher in men than in women.

**Table 1:** Clinical and metabolic characteristics of participants

	**Total**	**Men**	**Women**	**P**
**N (%)**	**1.145**	**689 (60.2%)**	**456 (39.8%)**	
Age (years)	66.8 ± 11.3	66.3 ± 11.4	67.4 ± 10.9	0.089
Duration diabetes (years)	9.1 ± 6.7	8.8 ± 6.5	9.4 ± 6.9	0.250
BMI (kg/m^2^)	30.3 ± 5.2	29.6 ± 4.5	31.1 ± 6.0	**0.001**
Obesity, n(%)	533 (46,5)	297 (43.1)	236 (51.8)	0.004
Abdominal waist circumference (cm)	100.0 ± 17.1	103.5 ± 12.1	100.2 ± 13.6	**0.001**
Smoking (n%)				
Yes	158 (13,8)	123 (17.9)	35 (7.7)	**0.001**
Ex-smoker	345 (30.1)	312 (45.2)	33 (7.2)	
Never	642 (56.1)	254 (36.9)	388 ( 85.1)	
HbA_1C_ (%)	7.3 ± 1.3	7.2 ± 1.2	7.3 ± 1.3	0.089
Hypertension (n%)	860 (75.1)	514 (74.6)	346 (75.9)	0.625
Systolic Blood Pressure (mmHg)	134.5 ± 13.2	134.3 ± 12.6	134.8 ± 14,0	0.984
Diastolic Blood Pressure (mmHg)	77.0 ± 9.1	77.1 ± 8.9	76.8 ± 9.3	0.477
Total cholesterol (mmol/L)	4.70 ± 0.93	4.63 ± 0.99	4.89 ± 0.90	**0.001**
LDL cholesterol (mg/dl)	2.79 ± 0,83	2.74 ± 0,86	2.84 ± 0,78	0.021
non-HDL cholesterol (mg/dl)	3.49 ± 0,91	3.46 ± 0,96	3.55 ± 0,90	0.086
HDL cholesterol (mg/dl)	1.29 ± 0.35	1.17 ± 0.31	1.33 ± 0.37	**0.001**
Triglycerides (mg/dl)	1.69 ± 0.96	1.70 ± 1.04	1.68 ± 0.87	0.450
Plasmatic creatinine (mg/dl)	0.93 ± 0.3	1.01 ± 0.3	0.8 ± 0.2	**0.001**
eGFR ml/min/1,73 m^2^	79.9 ± 23.5	80.4 ± 22.4	78.6 ± 23.6	0.142
Albumin/creatinine ratio (mg/g)	39.2 ± 144.3	46.3 ± 169.5	28.4 ± 93.4	0.979
Coronary heart disease (n%)	177 (15.4)	137 (19.9)	34 (7.5)	**0.001**
Heart failure (n%)	91 (7.9)	49 (7.1)	42 (9.2)	0.199
Peripheral vascular disease (n%)	98 (8.5)	85 (12.3)	13 (2.9)	**0.001**
Stroke (n%)	79 (6.9)	57 (8.3)	22 (4.8)	0.024

BMI = Body Mass Index. HbA_1C_ = glycosilated hemoglobin. eGFR = estimated glomerular filtration rate. LDL cholesterol = Low Density Lipoprotein cholesterol. HDL cholesterol = High Density Lipoprotein cholesterol.

Table 2 shows the prevalence of the different types of kidney disease. Prevalence of CKD in patients was 27.9% (CI 95% = 25.2 - 30.5), and the CKD prevalence was classified into the following stages: 3.5% with stage 1 (CI 95% = 2.3 - 4.6); 6.4% with stage 2 (CI 95% = 4.9 - 7.8); 16.8% with stage 3 (CI 95% = 14.6 - 19.0), including 11.6% with stage 3A (CI 95% = 9.7 - 13.5) and 5.2% with stage 3B (CI 95% = 3.9 - 6.5); and 1.2% with stages 4 and 5 (CI 95% = 0.4 - 1.7). Due to their low prevalence, stages 4 and 5 were analysed together. Prevalence for UACR ≥ 30 mg/g was 15.4% (CI 95% = 13.2 - 17.5), microlbuminuria 13% (CI 95% = 11.1 - 15), and macroalbuminuria 2.4% (CI 95% = 1.4 - 3.2). Renal impairment (RI) (eGFR <60) was found in 206 patients (18%) of whom 133 patients (64.6%) was stage 3A, 60 patients (29.1%) was stage 3B and 13 patients (6.3%) stages 4 and 5. Among patients with RI, 143 patients (69.4%) had normoalbuminuria.

**Table 2:** Prevalence of different types of chronic kidney disease in Spain

**eGFR,**	**Normoalbuminuria**	**Microalbuminuria**	**Macroalbuminuria**
**mL/min per 1.73 m**^**2**^	**ACR < 30 mg/g**	**ACR 30–300 mg/g**	**ACR > 300 mg/g**
≥ 90 (Stage 1)	298 (26%)	36 (3.14%)	4 (0.35%)
60-89.9 (Stage 2)	528 (46.1%)	68 (6%)	5 (0.45%)
45-59.9 (Stage 3A)	99 (8.65%)	26 (2.3%)	8 (0.7%)
30–44.9 (Stage 3B)	40 (3.5%)	15 (1.3%)	5 (0.45%)
< 30 (Stages 4–5)	4 (0.35%)	4 (0.35%)	5 (0.45%)
UACR	Normoalbuminuria	Microalbuminuria	Macroalbuminuria
N (%)	969 (84.6%)	149 (13.0%)	27 (2.4%)
eGFR ≥ 60			
mL/min per 1.73 m^2^	939 (82%)		
eGFR < 60			
mL/min per 1.73 m^2^	206 (18%)		
Any type of CKD	319 (27.9%)		

eGFR = estimated glomerular filtration rate. ACR = albumin-creatinine ratio. CKD = Chronic Kidney Disease.

The comparison of diabetic patients with CKD vs diabetic patients with no CKD showed no significant difference in their sex, BMI, weight, systolic arterial blood pressure (SABP), diastolic arterial blood pressure (DABP), tobacco use, or lipid profile values. No significant differences between the groups were found when comparing DM2 duration or glycaemia levels. However, diabetic patients with CKD had higher HbA1c values than diabetic patients without CKD. Diabetic patients with CKD had elevated creatinine values. Creatinine values, in CKD patients, increased as the disease progressed. Age was significantly correlated with CKD with increasing percentages of elderly patients in the most advanced stages. Patients diagnosed with arterial hypertension had a higher CKD prevalence (Table 3).

**Table 3:** Clinical characteristics of diabetic patients with any degree of CKD compared with diabetics with no CKD

	**no CKD**	**CKD 1**^**‘a’**^	**CKD 2**	**CKD 3A**	**CKD 3B**	**CKD 4-5**	**P**^**‘b’**^
	**FG ≥ 60**	**FG ≥ 90**	**FG 89-60**	**FG 59-45**	**FG 44-30**	**FG < 30**	
	**ACR < 30**	**ACR ≥ 30**	**CAC ≥ 30**				
N = 1145 (%)	826 (72.1)	40 (3.5)	73 (6.4)	133 (11.6)	60 (5.2)	13 (1.2)	
Age (years)	64.9 ± 10.8	62.8 ± 10.7	67.6 ± 11.1	74.4 ± 8.6	75.2 ± 10.0	73.6 ± 10.6	**0.001**
Gender (Men) (n%)	511 (61.9)	25 (62.5)	47 (64.4)	63 (47.4)	36 (60.0)	7 (53.8)	0.053
BMI (kg/m^2^)	30.1 ± 5.2	30.4 ± 4.4	30.4 ± 5.3	30.7 ± 5.1	30.9 ± 6.0	30.1 ± 5.1	0.985
Abdominal waist circumference (cm)	99.8 ± 16.5	101.7 ± 11.7	99.4 ± 19.3	99.8 ± 19.8	100.4 ± 19.1	105.5 ± 14.1	0.950
Smoking (n%)							
Yes	118 (14.3)	12 (30.0)	13 (17.8)	8 (6.0)	5 (8.3)	2 (15.4)	0.025
Ex-smoker	239 (28.9)	11 (27.5)	22 (30.1)	50 (37.6)	19 (31.7)	4 (30.8)	
Never	469 (56.8)	17 (42.5)	38 (52.1)	75 (56.4)	36 (60.0)	7 (53.8)	
HbA1_C_ (%)	7.2 ± 1.2	7.8 ± 1.6	7.7 ± 1.3	7.1 ± 1.2	7.0 ± 1.0	7.9 ± 1.9	**0.001**
Duration of DM (years)	8.5 ± 6.5	9.5 ± 6.6	9.7 ± 7.4	11.1 ± 8.5	11.0 ± 6.9	11.7 ± 8.7	0.386
Glycaemia (mmol/L)	8.05 ± 3.20	8.84± 3.23	9.00± 2.79	7.90± 2.63	7.48± 2.98	7.88 ± 3.40	0.002
Hypertension (n%)	578 (70.0)	32 (80.0)	60 (82.2)	121 (91.0)	56 (93.3)	13 (100)	**0.001**
Systolic Blood Pressure (mmHg)	134.0 ± 12.5	136.9 ± 15.9	136.6 ± 15.1	135.9 ± 14.2	134.2 ± 14.1	134.5 ± 16.9	0.610
Diastolic Blood Pressure (mmHg)	77.8 ± 8.7	79.3 ± 9.9	76.3 ± 9.6	74.2 ± 10.0	72.8 ± 8.8	71.7 ± 6.6	0.003
Dyslipidaemia (n%)	555 (67.2)	27 (67.5)	52 (71.2)	97 (72.9)	37 (61.7)	8 (61.5)	0.638
Total cholesterol (mmol/L)	4.75 ± 0.95	4.94 ± 1.12	4.78 ± 0.99	4.59 ± 0.89	4.65 ± 1.11	4.30 ± 0.91	0.113
LDL cholesterol (mmol/L)	2.81 ± 0.81	2.95 ± 1.00	2.76 ± 0.79	2.67 ± 0.85	2.64 ± 0.94	2.21 ± 0.56	0.082
non-HDL cholesterol (mmol/L)	3.50 ± 0.91	3.75± 1.07	3.60 ± 1.01	3.31 ± 0.91	3.45 ± 1.08	3.22 ± 0.94	0.064
HDL cholesterol (mmol/L)	1.24 ± 0.33	1.19 ± 0.33	1.18 ± 0.26	1.28 ± 0.43	1.17 ± 0.34	1.07 ± 0.25	0.244
Triglycerides (mmol/L)	1.63 ± 0.94	1.88 ± 0.94	1.89 ± 1.21	1.76 ± 1.03	1.90 ± 0.96	2.17 ± 1.07	0.477
Plasmatic creatinine (mg/dl)	0.8 ± 0.1	0.6 ± 0.1	0.9 ± 0.1	1.1 ± 0.1	1.5 ± 0.2	2.3 ± 0.5	**0.001**

^‘a’^ CKD (1, 2, 3A, 3B, 4–5) different stages of CKD ^‘b’^ P (Kruskall-Wallis test, Chi-squared test).

eGFR = estimated glomerular filtration rate. ACR = albumin-creatinine ratio. CKD = Chronic Kidney Disease. BMI = Body Mass Index. HbA_1C_ = glycosilated hemoglobin. eGFR = estimated glomerular filtration rate. LDL cholesterol = Low Density Lipoprotein cholesterol. HDL cholesterol = High Density Lipoprotein cholesterol.

The multivariate analysis by binomial logistic regression demonstrated that CKD was significantly associated with age. Older age was associated with higher CKD prevalence among women if SABP ≥ 150 mm Hg. Even at SAP ≥ 140 mm Hg, an increased CKD prevalence was observed. A previous clinical history of ischemic cardiac disease, cardiac insufficiency, or peripheral vascular disease was significantly associated with increased CKD prevalence (Table 4).

**Table 4:** Factors associated with CKD in patients with type 2 diabetes

	**OR (95% CI)**
Age (years)	
< 50	1
50-59	2.02 (0.93-4.41)
60-69	1.75 (0.82-3.76)
70-79	**3.24 (1.53-6.86)**
>80	**7.84 (3.50-17.54)**
Gender	
Men	1
Women	**1.36 (1.01-1.84)**
Systolic Blood Pressure (mmHg)	
< 130	1
130-139	0.85 (0.60-1.22)
140-149	1.01 (0.67-1.51)
≥ 150	**1.61 (1.03-2.53)**
Duration of DM 2 (years)	**1.02 (1.01-1.04)**
Coronary heart disease	**1.54 (1.04-2.28)**
Heart failure	**2.69 (1.64-4.40)**
Peripheral vascular disease	**2.71 (1.69-4.35)**

(CKD compared with “no CKD”).

CKD = Chronic Kidney Disease. OR = Odds Ratio. CI = Confident Interval.

## Discussion

To the best of our knowledge, this study was the first epidemiological investigation on CKD prevalence using the KDOQI criteria on Spanish patients attending primary care consults. Different studies have been performed in Spain on IR prevalence in DM2 patients [12-15]. The study by Lou Arnal et al. [16] has been the only report published on CKD prevalence in DM2 patients in primary care consults, and their study was performed with only one UACR measurement in Alcañiz, Teruel, Spain. The results of the PERCEDIME2 study showed an elevated CKD prevalence in DM2 patients in primary care consults in Spain. DM2 patients with CKD, 18% had a eGFR < 60 ml/min/1.73 m^2^ and 15.4% had albuminuria. In patients with a eGFR > 60 ml/min/1.73 m^2^, 9.9% presented albuminuria. In the study by Lou Arnal et al. [16], 34.5% of DM2 patients presented some stage of CKD, 16.1% of DM2 patients had albuminuria (14.3% microalbuminuria and 1.8% macroalbuminuria), and 9.4% of the patients with eGFR ≥ 60 ml/min/1.73 m^2^ had albuminuria. Vinagre et al. [17] found a prevalence of 20% for RI and 16.7% for albuminuria. In another study in Spain [8] the prevalence off different types of renal disease in DM2 was: 34,1% for any type of CKD, 22,9% RI and 19,5% for albuminuria. In a study performed in primary care centres in the Netherlands, van der Meer et al. [18] observed that 27.6% of DM2 patients had CKD and that 13.6% of these DM2 patients had albuminuria. Another study in the United Kingdom [19] showed that 31% of diabetic patients had a GFR < 60 ml/min/1.73 m^2^ and 37% of these patients had albuminuria. Pugliese et al. [20] found that the prevalence of RI was 18.7% and CKD 37.5% with the MDRD Study equation. Penno et al. [21] observed that in patients with renal impairment, as identified by an estimated glomerular filtration rate (eGFR) less than 60 ml/min per 1.73 m^2^, 56.6% were normoalbuminuric, 30.8% were microalbuminuric, and 12.6% were macroalbuminuric. In the United States Plantinga et al. [22] showed that 32.9% of U.S population had CKD and that 19.4% of these patients had albuminuria. In a study performed in Australia, Thomas et al. [23] observed that 23.1% of DM2 patients had a GFR < 60 ml/min/ 1.73 m^2^ and that 34.6% of these patients had albuminuria (27.3% microalbuminuria and 7.3% macroalbuminuria). In a Japanese study, Ohta et al. [24] found that 46% of DM2 patients had CKD and that 36.1% of DM2 patients had albuminuria, and this group reported that 25.2% of these patients had a GFR below 60 ml/min/1.73 m^2^. A study performed in Thailand [25] reported that DM2 patients in primary care consults had an albuminuria prevalence of 37.2% (26% microalbuminuria and 11.2% macroalbuminuria). In a study performed in Shanghai, Jia et al. [26] reported that 29.6% of DM2 patients had CKD and that 26.2% of these patients had albuminuria (22.8% microalbuminuria and 3.4% macroalbuminuria). Different prevalence of renal impairment and albuminuria among studies may due to the different methodology applied. Our study required that two of three determinations of eGFR or/and UACR, in a period of three or more months, should be presented normal or altered values.

Early detection of CKD has important clinical implications. It is well known that a diminished eGFR is associated with increased global mortality and cardiovascular episodes [2,27-29]. Different epidemiological studies have demonstrated that increased UAE is an important risk factor for CKD in DM2 patients. A meta-analysis of these studies [30] has shown that increased UAE doubles the cardiovascular morbidity and mortality risk. Moreover, UAE values in the high range of normality even predict cardiovascular and renal complications [1,31]. Patients with DM2 and CKD have an elevated risk of cardiovascular and renal complications, thus requiring improved intervention and control [32,33]. Albuminuria and mortality association is lineal in a logarithmic scale, and this risk doubles in patients with microalbuminuria with UACR levels within the optimal range independent of the eGFR and presence of other classic CVRF [5]. In the HUNT II study [34], the presence of albuminuria and reduced eGFR was associated with an increased risk of cardiovascular mortality. Astor et al. [35] reported that reduced eGFR and increased UAE independently increases the mortality risk for patients with CVRF, and the simultaneous occurrence of reduced eGFR and increased UAE was associated with an even higher risk. Our study found a high prevalence of PVD, different studies have shown a high prevalent PAD associated with microalbuminuria alone, reduced eGFR alone, and both reduced eGFR and microalbuminuria compared to those without microalbuminuria or reduced eGFR [36,37]. The higher prevalence in men may be related to the higher percentage of male smokers.

The present study had weaknesses and strengths. Although all autonomous communities were represented and efforts were made to ensure a representative distribution of the Spanish territory, this study was not a population-based study. The investigators were not randomly selected because only those willing to participate were involved in the study. The majority of these investigators were part of the RedGDPS (Red de Grupos de Estudio de la Diabetes en Atención Primaria de la Salud, Network for the Study of Diabetes in Primary Health Care). Thus, it was impossible to dismiss investigator-related bias and which patients were included. Another source of bias can come from the frequency of visits by the patients. However, the fact that the results of the present study were similar to other studies indicates that these biases had little influence on the final data. Importantly, all patients included in this study had to present normal or altered eGFR and UACR values in two of three determinations in a period of three or more months. If these requirements were not met, new eGFR and UACR determinations were required. The patients that did not fulfil this requisite were excluded from the final analysis.

## Conclusion

In conclusion, this study demonstrated that the prevalence of CKD in a sample of 1145 DM2 patients in primary care consults was 27.9%. A systematic determination of GFR and UACR would contribute to an earlier diagnosis, thus allowing intervention in early stages of the disease in which treatment is more efficacious.

## Abbreviations

UACR: Urine albumin-creatinine ratio;CKD: Chronic kidney disease;CVRF: Cardiovascular risk factor;DM2: Type 2 diabetes mellitus;eGFR: Estimated glomerular filtration rate;MDRD: Modification of diet in renal disease;ESRD: End-stage renal disease;UAE: Urinary albumin excretion

## Appendix I

Study investigators

Alum Bou A, Angullo Martínez E, Arnaiz Arroyo J, Artola Menendez S, Avila Lachica L, Barquilla García A, Barrot de la Puente J, Barutell Rubio L, Belinchón Sánchez M, Benito Badorrey B, Bobe Molina I, Bosch Costabella R, Buil Cosiales P, Carramiñana Barrera F, Carrillo Fernández L, Carrión Valero L, Casaseca Garcia P, Colt Loredana C, Comas Samper J, Cuevas Lopez J, De la Sen C, Díez Espino J, Domínguez Coello S, Domínguez Navarro D, Egocheaga Cabello I, Ferrer Menduiña X, Franch Nadal J, García Aparicio JM, Garcia Gallego F, García Soidán FJ, Garrido Redondo N, Gómez García C, Gomez Gonzalez L, Gonzalez Pastor C, Granero Fernandez E, Grau Bartomeu J, Guruchaga Arcelus M, Gutiérrez Almarza M, Gutierrez Perez A, Hernandez Monroy A, Hidalgo Ortiz M, Iglesias González R, Igual Fraile D, Llussà Arboix J, Marín Becerra T, Martín Manzano JL, Martínez Sierra C, Massana Raurich A, Massó Orozco J, Mata Cases M, Merino Pacho J, Millaruelo Trillo JM, Morales Escobar F, Modroño Freire M, Monzon Guerra A, Muguruza Valdeolmillos J, Mundet Tuduri X, Mur Marti T, Nogales Aguado P, Ortega Ríos FJ, Otero Serra X, Otzet Gramunt I, Pascual De la Pisa B, Patitucci Gómez F, Pinto Rivero M, Piulats Egea N, Pujol Martínez R, Quindimil Vázquez J, Robles Agüero E, Rodero Nuño M, Rodríguez Poncelas A, Ruiz Tamayo I, Sagredo Perez J, Sanchez Cabrero LG, Sangrós González FJ, Sanz Rebollo G, Sedano Garcia JI, Serra Laguarta M, Serrano Martin R, Socias Buades I, Torres Baile JL, Trenchs Rodriguez M, Ulibarri del Portillo J, Vazquez de Prada I, Vergara Fernández I, Villaro Gabarros M.

## Competing interest

The authors declare that there is no duality of interest associated with this manuscript.

## Authors’ contributions

AR-P, JD E and JGO contributed to the conception and design, ARP and JGO analysed and interpred the data. All authors contributed to the drafting of the article and gave final approval of the version to be published.

## Pre-publication history

The pre-publication history for this paper can be accessed here:

http://www.biomedcentral.com/1471-2369/14/46/prepub
